# An emerging role for *prdm* family genes in dorsoventral patterning of the vertebrate nervous system

**DOI:** 10.1186/s13064-015-0052-8

**Published:** 2015-10-24

**Authors:** Denise A. Zannino, Charles G. Sagerström

**Affiliations:** Department of Biochemistry and Molecular Pharmacology, University of Massachusetts Medical School, 364 Plantation St./LRB815, Worcester, MA 01605-2324 USA

**Keywords:** Neural tube, Dorsoventral patterning, Transcription, Neural progenitor, Prdm gene family

## Abstract

The embryonic vertebrate neural tube is divided along its dorsoventral (DV) axis into eleven molecularly discrete progenitor domains. Each of these domains gives rise to distinct neuronal cell types; the ventral-most six domains contribute to motor circuits, while the five dorsal domains contribute to sensory circuits. Following the initial neurogenesis step, these domains also generate glial cell types—either astrocytes or oligodendrocytes. This DV pattern is initiated by two morphogens—Sonic Hedgehog released from notochord and floor plate and Bone Morphogenetic Protein produced in the roof plate—that act in concentration gradients to induce expression of genes along the DV axis. Subsequently, these DV-restricted genes cooperate to define progenitor domains and to control neuronal cell fate specification and differentiation in each domain. Many genes involved in this process have been identified, but significant gaps remain in our understanding of the underlying genetic program. Here we review recent work identifying members of the *Prdm* gene family as novel regulators of DV patterning in the neural tube. Many Prdm proteins regulate transcription by controlling histone modifications (either via intrinsic histone methyltransferase activity, or by recruiting histone modifying enzymes). *Prdm* genes are expressed in spatially restricted domains along the DV axis of the neural tube and play important roles in the specification of progenitor domains, as well as in the subsequent differentiation of motor neurons and various types of interneurons. Strikingly, Prdm proteins appear to function by binding to, and modulating the activity of, other transcription factors (particularly bHLH proteins). The identity of key transcription factors in DV patterning of the neural tube has been elucidated previously (e.g. the *nkx*, *bHLH* and *pax* families), but it now appears that an additional family is also required and that it acts in a potentially novel manner.

## Introduction

Function of the adult central nervous system (CNS) relies on neural circuits to control activity. In order for such circuits to form, neurons must develop at the right time and place of the CNS during embryogenesis. A very elaborate genetic program is responsible for this process along both the head-to-tail (anteroposterior; AP) and back-to-front (dorsoventral; DV) axes of the CNS. In terms of the DV axis, secreted factors (Sonic hedgehog and Bone morphogenetic protein) initially establish gradients that are sensed by progenitor cells in the developing neural tube. Depending on their location in the gradient, different progenitor cells initiate the expression of different genes, leading to a pattern of gene expression along the DV axis. These genes subsequently refine the pattern by repressing each other’s expression, as well as by activating the expression of additional genes (e.g. neurotransmitters and their receptors) that define different types of neurons (e.g. GABAergic versus glutaminergic). Some genes involved in this process are known, but this review focuses on a new class of genes—the Prdm family—that appears to control gene expression during the formation of neurons along the embryonic DV axis.

## Review

### Prdm family proteins as regulators of gene expression

The Prdm family of proteins has only been recognized relatively recently (reviewed in [1, 2]). Proteins in this family are defined by an N-terminal PR domain, as well as by a varying number of zinc fingers (or, potentially, zinc knuckles). The PR domain was named after its initial identification in the *P*ositive regulatory domain I-binding factor 1 (formerly PRDI-BF1/Blimp-1, now Prdm1) and the *R*etinoblastoma protein-interacting zinc finger protein 1 (formerly Riz1, now Prdm2) factors [3–6]. While Prdm proteins may function differently in different contexts, emerging evidence suggest that these factors act to regulate gene expression.

The PR domain is related to the SET domain—a catalytic domain with histone lysine methyltransferase (HMT) activity named after the *S*u(var)3–9, *E*nhancer of zeste and *T*rithorax proteins—but the PR domain has diverged significantly from SET domains. In particular, most PR domains lack the H/RxxNHxC motif required for methyltransferase activity ([7]; reviewed by [1]). Accordingly, many Prdm proteins appear to lack intrinsic HMTase activity ([8–11] reviewed by [2]). Nevertheless, Prdm2, Prdm8, and Prdm9 have been reported to possess intrinsic HMT activity [2, 12–15], although the details of the catalytic mechanism are unclear. Strikingly, Prdm2 and Prdm8 methylate histone H3 on lysine 9 (H3K9), a modification associated with heterochromatin formation and transcriptional repression, whereas Prdm9 directs formation of H3K4me3—a modification associated with transcriptional activity [13–15]. Hence, Prdm proteins may mediate transcriptional activation or repression depending on the nature of their intrinsic HMT activity. Of the Prdm proteins that are enzymatically inactive, many are instead able to recruit histone-modifying enzymes and transcription regulatory factors via protein-protein interactions. Enzymes recruited in this manner include HMTs, the Polcycomb repressive complex 2 (PRC2), protein methyltransferase 5 (Prmt5), lysine specific demethylase 1 (Lsd1), as well as histone deacetylases (HDACs) and histone acetyltransferases (HATs) [10, 16–22] (reviewed in [1, 2]). For example, Prdm1, Prdm5, Prdm6 and Prdm12 all function with the G9a HMT [2, 8–10, 23] and Prdm3 with the Suv39H1 HMT [24] to methylate H3K9 and promote repression. Prdm1 can also function with Prmt5 to methylate H2AR3 and H4R3 [17]. Some Prdm family members require their zinc fingers for recruitment of histone modifying enzymes, while others (such as Prdm1 and Prdm3) also make use of a proline-rich domain [1, 25, 26]. Additionally, transcriptional regulators can be recruited by Prdm proteins, such as the recruitment of Groucho by Prdm1, and the recruitment of CtBP by Prdm2, Prdm3 and Prdm16 ([27–34], reviewed [1]). Hence, Prdm proteins appear to function by modulating gene expression states either directly (via intrinsic HMTase activity), or indirectly (via recruitment of various cofactors).

In order to affect gene expression, Prdm proteins need to access genomic sites in chromatin. Accordingly, Prdm1, Prdm3, Prdm5, Prdm9, Prdm13, Prdm14, and Prdm16 bind DNA directly in a sequence dependent manner via their zinc-finger domains ([9, 35–43] reviewed in [1, 2]). While many Prdm proteins have only been tested for DNA binding using in vitro systems, ChIP-seq experiments (chromatin immunoprecipitation using Prdm-specific antibodies followed by deep sequencing) have also identified genomic binding sites for a subset of Prdm factors (Prdm1, Prdm3, Prdm13, and Prdm14) [35, 37, 41, 43–46]. Prdm members that do not bind DNA directly instead appear to utilize binding partners to indirectly associate with DNA, as in the case of Prdm8 accessing DNA by binding together with Bhlhb5 in the developing nervous system [47] and Prdm16 binding with C/EBPβ to promote brown adipose tissue [48]. Again, the zinc finger motifs, as well as proline-rich domains and zinc knuckles, are likely to mediate binding of Prdm proteins to partner proteins to facilitate access to genomic sites. Based on their association with DNA (directly or indirectly), as well as their ability to modify histones (directly or indirectly) and recruit transcriptional regulators, it is likely that Prdm family proteins function to regulate gene expression states. Indeed, Prdm factors appear capable of activating or repressing target genes depending on the specific context—as reported for Prdm1 and Prdm2 [49, 50]. Prdm proteins have been reported to function in numerous settings, including hematopoiesis, adipogenesis and the maintenance of stem cell identity (reviewed by [1, 2]). More recently, several studies have indicated a central role for Prdm factors in the establishment of neuronal cell fates, particularly in the forming hindbrain and spinal cord.

### Multiple roles for Prdm proteins in dorsoventral patterning of the neural tube

**Fig. 1:**
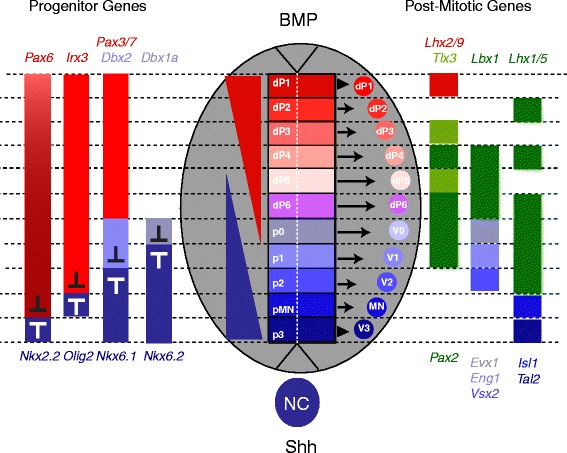
Schematic diagram of a neural tube cross section. Dorsoventral domains are established by opposing concentration gradients of Shh and BMP (center), which regulate progenitor gene expression (left). The progenitor genes cross-repress each other to establish domain boundaries. Each domain will give rise to a specific cell type that expresses various post-mitotic differentiation genes (right)

Shortly after neural tube closure, the neuroepithelium undergoes extensive transformations, including cell proliferation and specification, to give rise to various neuronal and glial cell types necessary for motor and sensory circuits. This process requires several steps (Fig. 1): First, gene expression is initiated along the dorsoventral (DV) axis of the neural tube in response to morphogen gradients. Second, these domains are refined and discrete gene expression boundaries established by complex regulatory interactions among many genes. Third, distinct neuronal and glial cell types are specified and differentiate from each progenitor domain. Strikingly, emerging data suggest that each of these steps may be under the control, at least in part, of *Prdm* family genes (Table 1).

**Table 1:** Summary of *Prdm* gene expression and function in the nervous system

Prdm gene	Nervous system expression	Nervous system function	Intrinsic HMT activity	Direct DNA binding	References
Prdm1	CNS: photoreceptors	CNS: photoreceptor identity	No	Yes	[8, 108, 109, 120, 144–147]
	PNS: prechordal plate, branchial arches, Rohon-Beard neurons	PNS: branchial arch formation, Rohon-Beard specification			
Prdm3	CNS: telencephalon, tegmentum, diencephalon, hindbrain	CNS: olfactory receptor development		Yes	[28, 34, 120, 148]
	PNS: branchial arches	PNS: craniofacial development			
Prdm4	CNS: cerebral cortex	CNS: in vitro neural stem cell proliferation and differentiation	Yes		[149–151]
Prdm5	CNS: ventral spinal cord	PNS: development of the neuocranium		Yes	[9, 152, 153]
	PNS: neurocranium				
Prdm6	CNS: spinal cord neurons		Yes	Yes	[74, 154]
Prdm8	CNS: telencephalon, retina, tegmentum, cerebellum, hindbrain and spinal cord	CNS: axonal outgrowth, neocortical neuron morphology	No	Yes	[15, 47, 72, 74, 120, 155]
Prdm10	PNS: neural crest	CNS: primary dendrite initiation			[156, 157]
Prdm12	CNS: telencephalon, tegmentum, cerebellum, midbrain, hindbrain and spinal cord p1 domain	CNS: formation of V1 interneurons, pain perception and sensory neuron development			[73, 74, 81, 120, 158, 159]
	PNS: cranial placodes				
Prdm13	CNS: tegmentum, hindbrain, spinal cord, retina	CNS: GABAergic interneuron development		Yes	[43, 74, 80, 120]
Prdm14	CNS: ventral spinal cord	CNS: CaP motor neuron axonal projection		Yes	[46, 74, 120]
Prdm16	CNS: forebrain, telencephalon, hindbrain, retina	CNS: olfactory neuron development	Yes	Yes	[28, 34, 74, 120, 160]
	PNS: craniofacial structures	PNS: craniofacial development			

Blank cells indicate categories were information is lacking in the literature. The list of expression domains and functions is not exhaustive

#### *Prdm* genes are expressed in discrete domains along the DV axis of the neural tube

**Fig. 2:**
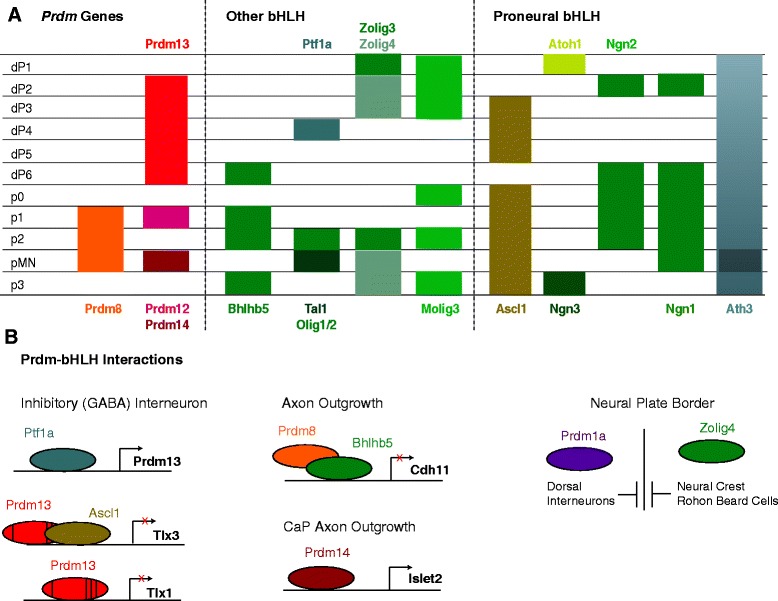
Summary of Prdm expression domains and interactions. **a**. Schematic representation of *Prdm* and *bHLH* expression domains along the dorsoventral axis of the neural tube. **b**. Diagrams of interactions between *Prdm* and *bHLH* genes discussed in the text. Left panel: The *Prdm13* gene is regulated by Ptf1a. Additionally, Prdm13 regulates both *Tlx1* and *Tlx3* expression – in the latter case Prdm13 acts in a complex with Ascl1. Middle panel (top): Prdm8 acts in a complex with Bhlhb5 to regulate *Cdh11* expression. Middle panel (bottom): Prdm14 binds the promoter region of *Islet2* to regulate its expression. Right panel: Prdm1a and zebrafish Olig4 cross repress each other’s expression at the neural plate boundary

Studies in several vertebrate species have demonstrated a critical role for Sonic Hedgehog (Shh) in patterning of the ventral neural tube and in specification of ventral neuronal cell types. Specifically, Shh is a morphogen secreted from the notochord and floor plate that—along with factors such as Chordin and Noggin that oppose the dorsally expressed BMP morphogen (see below)—induces gene expression in the ventral neural tube (reviewed by [51–53]). This has been demonstrated experimentally by overexpression of Shh in vivo and by application of exogenous Shh to neural tube explants in culture, as well as by inhibiting Shh signaling using neutralizing antibodies or germ line knock outs [54–63]. The Shh gradient subdivides the neural tube into distinct DV progenitor domains by regulating the expression of different genes at different thresholds of Shh signaling ([64–68]; Fig. 1). In particular, Shh activates genes such as *Nkx6.1*, *Nkx6*.2, *Nkx2.2*, and *Olig2*, while it represses genes such as *Pax3*, *Pax6*, *Pax7*, *Dbx1*, *Dbx2* and *Irx3* [63–71]. Notably, at least three *Prdm* genes (*Prdm8*, *Prdm12*, and *Prdm14*; Fig. 2) are expressed in the ventral neural tube. Expression of *Prdm8* is present in the p1, p2 and pMN domains [72], while *Prdm14* is expressed in the pMN domain, specifically in a subset of motor neurons—the Caudal Primary (CaP) motor neurons [46]—and *Prdm12* is expressed in the p1 domain [73, 74]. Based on their expression domains, these three *Prdm* genes are likely to be regulated by Shh signaling. Indeed, treatment with cyclopamine (a Shh signaling inhibitor), causes a reduction of *Prdm12b* expression in zebrafish [73]. This suggests that *Prdm12b* is partially dependent on Shh signaling, as previously reported for other genes expressed in the p1 domain [67], but it remains to be determined if *Prdm8* and *Prdm14* expression is also regulated by Shh signaling.

Similar to Shh signaling in the ventral neural tube, Bone Morphogenetic Proteins (BMPs) function in the dorsal neural tube to pattern progenitor domains and regulate cell specification (Fig. 1). In particular, BMP4, BMP5 and BMP7, as well as the related Gdf7 protein, are expressed in the ectoderm overlaying the neural tube and function in concentration gradients to establish the dP1-6 progenitor domains [75, 76]. As expected, increasing or decreasing the BMP signal in the dorsal neural tube expands or reduces the specification of dorsal cell types, respectively [77–79]. In addition, loss of BMP receptors leads to loss of the dP1 and a dorsal shift in the dP2 domain [79], while expression of a constitutively active BMP receptor causes a ventral shift in *Pax7* expression and an up-regulation of the dP1 expressed *Atoh1 (*previously *Math1)* [77]. Notably, *Prdm13* is expressed in the dorsal neural tube in the dP2-dP6 domains ([43, 80]; Fig. 2), suggesting that it may be regulated by BMP. However, *Prdm13* has been shown to act downstream of *Ptf1a*, so BMP may function indirectly to control *Prdm13* expression [43, 80]. Notably, the expression of *Prdm12b* in the p1 domain may also be sensitive to BMP signaling since the p0 and p1 domains are dependent on both Shh and BMP signaling (e.g. *Evx1* and *En1* expression in p0/p1 is reduced upon introduction of a constitutively active BMP receptor; [77]; reviewed by [52]). Accordingly, *Prdm12* is regulated by BMP signaling outside the neural tube, such as in pre-placodal ectoderm [81].

Factors in addition to Shh and BMP are also involved in establishing progenitor domains in the neural tube. For instance, ventrally expressed BMP inhibitors (*Chordin*, *Noggin* and *Follistatin*) are required to suppress BMP signaling, thereby promoting the formation of ventral progenitor domains [82–87]. FGF signaling also promotes ventral fates by repressing *Pax6*, *Irx3*, *Dbx1* and *Dbx2* [88–90]. In contrast, *Wnt1* and *Wnt3a* expressed in the roof plate are required for formation of dorsal progenitor domains (reviewed in [52, 53]), as loss of Wnt signaling leads to reduction in dP1 and dP2 neurons, with excess formation of dP3 neurons [91]. Retinoic acid (RA) is also released from the roof plate [92] to promote formation of dorsal progenitor domains. Accordingly, reduced RA signaling leads to dorsal expansion of ventral genes such as *Nkx6.1* and *Nkx2.2* [90, 93, 94]—although this may be a partially indirect effect mediated by loss of *Pax6* [52]—and reduced expression of dorsal genes such as *Bmp4/7*, *Msx2*, *Pax3/7*, *Wnt1/3a*, *Pax6* and *Irx3* [90, 94–97]. Several *Prdm* genes are regulated by these pathways outside of the neural tube. For instance, expression of *Prdm12* in *Xenopus* lateral pre-placodal ectoderm is reduced when *Wnt3a* is over-expressed [81] and *Prdm14* expression in primordial germ cell specification may be activated when *T-Brachyury—*a downstream target of *Wnt3a*—binds to an enhancer at the *Prdm14* gene [98]. Furthermore, RA treatment induces expression of *Prdm12* in cell lines [23]. Hence, it is plausible that *Prdm* gene expression is induced by Fgf, Wnt and/or RA signaling also in dorsoventral patterning of the neural tube.

#### *Prdm* genes are involved in mutually repressive interactions between gene expression domains

The distinct boundaries observed between progenitor domains in the neural tube are established by cross-repressive interactions between adjacent gene expression domains (Fig. 1). Several mutually repressive pairs of transcription factors have been identified, including *Pax6/Nkx2.2*, *Dbx2/Nkx6.1* and *Irx3/Olig2* ([64, 66, 68–70, 99–102]; reviewed in [53]). For instance, *Irx3* and *Olig2* repress each other’s expression, thereby setting up the p2/pMN boundary [69, 102]. Accordingly, knock-out of *Olig2* causes a ventral expansion of *Irx3* and leads the pMN domain to adopt more dorsal characteristics. Hence, this domain gives rise to V2 interneurons and astrocytes instead of the motor neurons and oligodendrocytes that normally arise from the pMN domain [102]. Given the expression of *Prdm* genes in discrete domains along the dorsoventral axis of the neural tube, it is likely that *Prdm* genes also engage in mutually repressive interactions. For instance, *Prdm12b* is expressed in the p1 progenitor domain and shares an expression boundary with *Nkx6.1*—which is expressed in the p2, pMN and p3 domains—at the p1/p2 border. Notably, loss of *Prdm12b* function leads to ectopic expression of *Nkx6.1* dorsally [73], suggesting that *Prdm12b* represses *Nkx6.1* expression. However, it is not clear if this effect is direct, nor is it clear if *Nkx6.1* reciprocally represses *Prdm12b* expression. Furthermore, zebrafish *Olig4* (*Olig3* in mouse) is expressed in the dP1-3 domains, where it is required for the specification of dorsal interneurons [103–105], whereas *Prdm1a* is expressed adjacent to *Olig4* at the neural plate border [106]. Knockdown of *Olig4* results in a severe reduction, or loss, of dorsal interneurons and a corresponding increase in cell types normally specified by *Prdm1a—*neural crest cells and Rohon-Beard cells [103, 105, 107–109]. Further studies confirmed that *Prdm1a* represses *Olig4* expression, and vice versa, to establish and maintain the neural plate border and interneuron domains [106]. As *Prdm* gene function in the neural tube becomes analyzed more closely, it is likely that additional cases of reciprocal repression will be identified.

#### *Prdm* genes regulate neuronal specification and differentiation in the neural tube

Through their roles as regulators of gene expression, Prdm family proteins affect the specification and differentiation of neuronal subtypes from various progenitor domains.

#### *Prdm12b* is required for the formation of V1 interneurons

Prdm12 was originally described in chronic myeloid leukemia as a gene located in a deleted region on chromosome 9 [110, 111]. *Prdm12* also plays a role controlling proliferation in various cell lines [23]. Expression of *Prdm12* within the developing CNS was first described in the mouse, with expression domains identified in the ventricular zone of the telencephalon, as well as in distinct domains within the hindbrain and spinal cord [74]. A similar pattern is observed in the zebrafish neural tube—specifically, *Prdm12b* expression is limited to the p1 domain in the hindbrain and spinal cord, as well as to cells adjacent to the exit points of the ventral motor roots [73]. The spinal cord p1 domain gives rise to V1 interneurons, a class of inhibitory glycinergic interneurons that function to regulate motor circuits controlling trunk and tail musculature [112–117] and reviewed ([118]). V1 interneurons are defined by their expression of the *Eng1* gene [64, 115]. Disruption of *Prdm12b* function leads to loss of *Eng1b* positive cells in zebrafish hindbrain and spinal cord, suggesting that *Prdm12b* is required for V1 interneuron formation. Strikingly, fish lacking *Prdm12b* function, and therefore also lacking V1 interneurons, display a defective escape response. In particular, when control fish are touched on the head, they bend their body into a single C-turn—bringing their head adjacent to the tail and orienting the head away from the stimulus—and then swim away. In contrast, *Prdm12b*-deficient fish exhibit multiple C-turns, display a longer response time with less productive swimming movements, and take longer between alternating body bends [73]. Hence, *Prdm12b* is required for the formation of the p1 domain and p1-derived neurons, although it remains unclear if the behavioral defect results from the loss of V1 interneurons in spinal motor circuits, or from the loss of some other class of p1-derived neurons in the hindbrain.

#### *Prdm14* controls formation of motor neuron axons

The pMN domain gives rise to motor neurons in a process that appears to require *Prdm14*. In zebrafish, four types of primary motor neurons (one of which is transient) are generated in the spinal cord, including CaP (caudal primary), MiP (middle primary), RoP (rostal primary) and VaP (variable primary). A zebrafish mutant for *Prdm14*, named *short lightning (slg)*, was identified in a gene-trap screen using the *Tol2* transposon system when a transposon inserted into the *Prdm14* locus [46]. Strikingly, loss of *Prdm14* does not affect the specification of motor neurons. Instead, CaP motor neurons in *slg* embryos display shortened axons and such embryos exhibit impaired escape responses and diminished swimming movements [46]. Prdm14 binds DNA via its zinc finger domain [41] and has been shown to occupy binding sites upstream of the *Islet2* gene [46], which is required for the development of motor neurons. Notably *Prdm14* is expressed in CaP and VaP motor neurons, but not in MiP or RoP motor neurons. Similarly, *Islet2* is restricted to CaP and VaP, while *Islet1* is maintained in MiP and RoP, motor neurons. Hence, *Prdm14* and *Islet2* are co-expressed in CaP motor neurons, explaining why the defects in *slg* mutants are restricted to this cell type. Interestingly, *Prdm14* and *Islet2* are also co-expressed in Rohon-Beard cells (a class of primary sensory neurons found in zebrafish), but *Prdm14* does not regulate *Islet2* expression in this cell population. Instead, another Prdm gene, *Prdm1a*, is expressed in Rohon-Beard cells where it regulates *Islet2* [46, 119]. Thus, *Prdm14* regulates *Islet2* in CaP motor neurons and *Prdm1a* regulates *Islet2* in Rohon-Beard cells, illustrating two examples of *Prdm* genes controlling neuronal cell fate. We note that *Prdm8* is also expressed in the pMN domain, but apparently not in precursors of motor neurons [72] and it is therefore unlikely to control motor neuron formation.

#### *Prdm13* controls formation of GABAergic neurons

*Prdm13* is expressed in the dP6-dP2 progenitor domains of the dorsal spinal cord [34, 43, 74, 80, 120]. *Prdm13* is both necessary and sufficient to promote differentiation of inhibitory (GABAergic) neurons over excitatory (glutamatergic) neurons [43, 80]. Specifically, *Prdm13* represses expression of *Tlx1* and *Tlx3* (excitatory lineage genes) by directly binding to their regulatory regions, as well as by binding to the Ascl1 transcription factor and inhibiting its ability to activate *Tlx3* expression (see below for further details; [43]). Furthermore, *Prdm13* blocks the ability of Neurogenin2 (another transcription factor involved in neuronal specification; [121, 122]) to activate transcription of *Tlx3* [80].

#### *Prdm8* controls targeting of projection neurons in the telencephalon

*Prdm8* is expressed at multiple sites of the CNS, including the dorsal telencephalon and the pMN-p1 domains of the hindbrain and spinal cord. Loss of function analyses in the mouse revealed that *Prdm8* is required for proper targeting of several major axon tracts (corticospinal tract, hippocampal commissure, anterior commissure and corpus callosum), apparently by cooperating with the *Bhlhb5* gene (see below for further details; [47]).

### Prdm family proteins form complexes with other transcription factors to control gene expression

While it appears clear that Prdm family proteins act as transcription factors to control neuronal differentiation, it remains unclear precisely how they function. For instance, Prdm12b regulates expression of *Eng1b* in V1 interneurons, but it is not clear that Prdm12b binds DNA. Furthermore, Prdm1a, Prdm12b, Prdm13 and Prdm14 all control transcription, but these proteins do not all contain recognizable transcription regulatory domains. The simplest explanation would be that Prdm proteins act in complexes with other regulatory factors. Indeed, there are now several reports of Prdm proteins interacting physically with other transcription factors in larger complexes.

#### *Prdm13* interacts with Ascl1 to promote formation of GABAergic neurons

As discussed, *Prdm13* is expressed in the dP2-dP6 progenitor domains [34, 43, 74, 80, 120], but appears to function primarily in dP4. In this region of the neural tube, several bHLH proteins function together with various binding partners in a combinatorial code to specify individual cell fates (reviewed by [123]). Specifically, dP1, dP2, dP3 and dP5 give rise to excitatory (glutamatergic) neurons, while dP4 gives rise to inhibitory (GABAergic) neurons. The bHLH transcriptional activators *Ascl1*, expressed in dP3-5, and *Ptf1a*, expressed only in dP4, are required for the formation of excitatory versus inhibitory interneurons in dP3-dP5, such that *Ascl1* alone drives expression of the *Tlx1* and *Tlx3* genes to promote excitatory interneuron fates in dP3 and dP5, while co-expression of *Ptf1a* with *Ascl1* in dP4 promotes inhibitory interneuron fates by repressing *Tlx1* and *Tlx3* transcription and promoting expression of *Pax2* and *Lbx1* [122, 124–133]. Strikingly, it appears that *Ptf1a* acts via *Prdm13* in dP4 to switch Ascl1 from a transcriptional activator to a repressor. In particular, Ptf1a directly activates *Prdm13* expression in dP4 and Prdm13 binds the same regulatory regions as Ascl1 at the *Tlx3* gene [43, 80]. Furthermore, Prdm13:Ascl1-containing complexes can be detected by co-immunoprecipitation [43], suggesting that such complexes regulate *Tlx3* expression. Prdm13 also interferes with the ability of Neurog2 to activate *Tlx3* [80], but it is not clear if this involves the formation of a complex between Prdm13 and Neurog2. Lastly, Prdm13 represses *Tlx1* in the absence of Ascl1 [43], suggesting that Prdm13 may also be a transcriptional repressor in its own right, or that it may interact with other factors in the regulation of *Tlx1*.

Prdm13 has been reported to exhibit methyltransferase activity [80], but it is not clear if this activity is intrinsic to Prdm13, or the result of a co-purifying factor. Indeed, the Prdm13 PR domain—the domain with sequence similarity to methyltransferases—is not required for its ability to repress *Tlx1* and *Tlx3* [43], indicating that intrinsic methyltransferase activity is unlikely to be required for Prdm13 to function as a repressor. In contrast, the Prdm13 zinc fingers are required for it to function as a repressor [43].

Notably, *Prdm13* expression overlaps with the expression domains of other bHLH genes and it is therefore possible that additional Prdm13:bHLH complexes may form. For instance, *Prdm13* expression overlaps with *Olig3* (*Olig4* in zebrafish) expression in dP2 and dP3 [104, 105, 134, 135]. The dP2 and dP3 domains give rise to Class A interneurons and loss of *Olig3* function re-specifies them to produce dP4 interneurons [104, 135]. Given the physical interaction between Prdm13 and the bHLH factor Ascl1 in dP4, this raises the possibility that Prdm13 and Olig3 could function as a complex in the specification of dP2 and dP3, but this remains to be explored.

#### *Prdm8* acts in a complex with Bhlhb5 to control neural circuit assembly

The *Bhlhb5* gene is closely related to the *Olig* subfamily of bHLH genes, but is expressed in postmitotic neurons—particularly in excitatory neurons of the dorsal telencephalon [136, 137, 138]. Similar to the Olig proteins, *Bhlhb5* appears to act as a transcriptional repressor [139, 140]. *Bhlhb5* mutant mice exhibit axonal projection defects such that axons originating in the dorsal telencephalon fail to reach their targets (Joshi 2008). This phenotype is shared with *Prdm8* mutant mice such that both mutants exhibit mis-targeting of the main fiber tracts connecting the cerebral hemispheres [47]. Importantly, *Bhlhb5* and *Prdm8* are co-expressed in many populations of differentiating neurons, including the dorsal telencephon, indicating that they may function together. Indeed, further analyses revealed that Bhlhb5 and Prdm8 proteins interact in a co-immunoprecipitation assay and that the two proteins co-occupy promoter elements in vivo, as defined by ChIP analysis [47]. Strikingly, the same set of target genes is up-regulated in *Bhlhb5* and *Prdm8* mutants, though the mutants differ such that Bhlhb5 can bind targets in the absence of Prdm8—but not vice versa. Hence, it appears that Bhlhb5 binds DNA directly (most likely as a homodimer via a canonical E-box motif), but cannot repress target genes in the absence of Prdm8, while Prdm8 is a repressor that cannot access target genes in the absence of Bhlhb5. Among the Bhlhb5/Prdm8 target genes, *Cdh11* is expressed in several intermediate targets of the corticospinal projections and is up-regulated in *Bhlhb5* and *Prdm8* mutant mice. Analysis of *Bhlhb5/Cdh11* double mutants, which allows reduction of *Cdh11* expression in the *Bhlhb5* mutant background, revealed that axonal targeting was partially rescued [47], suggesting that Bhlhb5/Prdm8 regulates neuronal circuit formation at least in part by controlling *Cdh11* expression levels.

*Bhlhb5* and *Prdm8* are co-expressed at other sites in the CNS. For instance, both genes are expressed in the spinal cord p2 domain [72, 141, 142] and *Bhlhb5* has been implicated in specifying V2a over V2b interneurons [141], suggesting that Bhlhb5:Prdm8 complexes may act also in V2a differentiation. Furthermore, *Bhlhb5* expression overlaps with the expression of other Prdm genes—such as *Prdm12* in the p1 domain and *Prdm13* in the dP6 domain—and *Bhlhb5* is involved in the specification of interneurons from those domains [141, 142]. While this suggests potential interactions for Prdm12 and Prdm13 with Bhlhb5, this remains to be tested.

## Conclusions

### Emerging principles for *Prdm* function in the developing CNS

Embryogenesis is replete with transcription factor “codes” and networks working in concert to specify and differentiate various cell types. Here we have reviewed the function of *Prdm* genes expressed within the neural tube, discussed the known interactions between bHLH transcription factors and Prdm family members, as well as proposed additional processes where members of these families are expressed, function, and may directly interact. From this review, some general principles are beginning to emerge. First, many *Prdm* family genes function in the developing CNS. To date, five *Prdm* genes (*Prdm1a, Prdm8, Prdm12b, Prdm13* and *Prdm14*) have been shown to control CNS development. Second, *Prdm* genes are involved in multiple aspects of CNS development. *Prdm12b* and *Prdm1a* play roles in early patterning by controlling the formation of expression domain boundaries (*Prdm12b* controls the p1/p2 boundary and *Prdm1a* the neural plate border; [73, 106]), while *Prdm13* acts on cell fate decisions to control the formation of inhibitory (GABAergic) over excitatory (glutamatergic) neurons [43, 80]. In contrast, *Prdm14* acts during motor neuron maturation to control proper axonal outgrowth [46] and *Prdm8* acts to control appropriate axonal targeting during neural circuit formation [47]. Third, Prdm proteins function in complexes with other transcription factors. In particular, Prdm8 functions by forming a repressor complex with Bhlhb5 in the dorsal telencephalon [47] and Prdm13 interacts with Ascl1 to promote formation of GABAergic neurons [43, 80]. These findings suggest a general model where Prdm family members function in multi-protein transcription regulatory complexes that control diverse aspects of neural development—from the patterning of expression domains and cell specification to axonal projections and circuit formation.

Since the *Prdm* family is still relatively poorly characterized and new members continue to be added, it is likely that additional *Prdm* genes are involved in CNS development—or that known *Prdm* genes will have additional functions. As discussed, Prdm13 physically interacts with the bHLH protein Ascl1 in the dP4 domain [43], but *Prdm13* is also co-expressed with another bHLH protein—*Olig3* (*Olig4* in zebrafish)—in the dP1-dP3 domains, suggesting that Prdm13:Olig3(4) complexes may act in dP1-dP3. Similarly, both Prdm12b and bHLHb5 are expressed in the p1 domain and play roles in V1 interneuron specification [73, 74, 141, 142], indicating they might interact in a complex. Perhaps even more compelling, Bhlhb5 and Prdm8—that are known to interact in the telencephalon—are also co-expressed in the p2 domain (where Bhlhb5 has a known role in V2a interneuron specification [141, 142]) suggesting that they may act together in a complex also in the p2 domain.

There are several gene families with important roles in early neural development. In particular, the bHLH, Pax, Dbx, and Nkx families regulate neuronal cell fate specification and differentiation [52, 53, 123, 143]. The data reviewed here demonstrate that *Prdm* genes also have essential functions in CNS development, thereby placing the Prdm family alongside these other gene families as key regulators of neural development. Strikingly, there appears to be a particularly close relationship between the bHLH and Prdm families (Fig. 2b) with Prdm proteins having the ability to modulate bHLH protein function via the formation of protein complexes (e.g. Prdm8 binding with Bhlhb5 [47] and Prdm13 binding with Ascl1 [43]).

## Abbreviations

CNS: Central nervous system
DV: Dorsoventral
AP: Anteroposterior
HMT: Histone methyltransferase
HDAC: Histone deacetylase
HAT: Histone acetyl transferase
ChIP: Chromatin immunoprecipitation
CaP: Caudal primary
MiP: Middle primary
RoP: Rostral primary
VaP: Variable primary
